# Added Value of Radiotherapy Following Neoadjuvant FOLFIRINOX for Resectable and Borderline Resectable Pancreatic Cancer: A Systematic Review and Meta-Analysis

**DOI:** 10.1245/s10434-021-10276-8

**Published:** 2021-06-17

**Authors:** Quisette P. Janssen, Jacob L. van Dam, Isabelle G. Kivits, Marc G. Besselink, Casper H. J. van Eijck, Marjolein Y. V. Homs, Joost J. M. E. Nuyttens, Hongchao Qi, Hjalmar J. van Santvoort, Alice C. Wei, Roeland F. de Wilde, Johanna W. Wilmink, Geertjan van Tienhoven, Bas Groot Koerkamp

**Affiliations:** 1 Department of Surgery, Erasmus MC Cancer Institute, Rotterdam, The Netherlands; 2 Department of Surgery, Cancer Center Amsterdam, University of Amsterdam, Amsterdam, The Netherlands; 3 Department of Medical Oncology, Erasmus MC Cancer Institute, Rotterdam, The Netherlands; 4 Department of Radiation Oncology, Erasmus MC Cancer Institute, Rotterdam, The Netherlands; 5 Department of Biostatistics, Erasmus MC University Medical Center, Rotterdam, The Netherlands; 6 Department of Surgery, Regional Academic Cancer Center Utrecht, St. Antonius Hospital and University Medical Center Utrecht, Nieuwegein, The Netherlands; 7 Department of Surgery, Memorial Sloan Kettering Cancer Center, New York, NY USA; 8 Department of Medical Oncology, Cancer Center Amsterdam, University of Amsterdam, Amsterdam, The Netherlands; 9 Department of Radiation Oncology, Cancer Center Amsterdam, University of Amsterdam, Amsterdam, The Netherlands

## Abstract

**Background:**

The added value of radiotherapy following neoadjuvant FOLFIRINOX chemotherapy in patients with resectable or borderline resectable pancreatic cancer ((B)RPC) is unclear. The objective of this meta-analysis was to compare outcomes of patients who received neoadjuvant FOLFIRINOX alone or combined with radiotherapy.

**Methods:**

A systematic literature search was performed in Embase, Medline (ovidSP), Web of Science, Scopus, Cochrane, and Google Scholar. The primary endpoint was pooled median overall survival (OS). Secondary endpoints included resection rate, R0 resection rate, and other pathologic outcomes.

**Results:**

We included 512 patients with (B)RPC from 15 studies, of which 7 were prospective nonrandomized studies. In total, 351 patients (68.6%) were treated with FOLFIRINOX alone (8 studies) and 161 patients (31.4%) were treated with FOLFIRINOX and radiotherapy (7 studies). The pooled estimated median OS was 21.6 months (range 18.4–34.0 months) for FOLFIRINOX alone and 22.4 months (range 11.0–37.7 months) for FOLFIRINOX with radiotherapy. The pooled resection rate was similar (71.9% vs. 63.1%, *p* = 0.43) and the pooled R0 resection rate was higher for FOLFIRINOX with radiotherapy (88.0% vs. 97.6%, *p* = 0.045). Other pathological outcomes (ypN0, pathologic complete response, perineural invasion) were comparable.

**Conclusions:**

In this meta-analysis, radiotherapy following neoadjuvant FOLFIRINOX was associated with an improved R0 resection rate as compared with neoadjuvant FOLFIRINOX alone, but a difference in survival could not be demonstrated. Randomized trials are needed to determine the added value of radiotherapy following neoadjuvant FOLFIRINOX in patients with (B)PRC.

**Supplementary Information:**

The online version contains supplementary material available at 10.1245/s10434-021-10276-8.

Pancreatic ductal adenocarcinoma is one of the most aggressive solid tumors.^1^ Although it is only the 12th most common cancer globally, it is one of the leading causes of cancer-related death in developed countries.^2^ Around 20–30% of patients have resectable or borderline resectable pancreatic cancer [(B)RPC] at diagnosis. In the most recent National Comprehensive Cancer Network (NCCN) and American Society of Clinical Oncology (ASCO) guidelines, neoadjuvant treatment is recommended for patients with BRPC. For patients with resectable tumors, neoadjuvant treatment is considered an alternative to upfront surgery, especially in patients with biochemical findings suggesting systemic disease (e.g., elevated tumor markers).^3^–^5^

In the past two decades, numerous studies on neoadjuvant chemoradiotherapy for pancreatic cancer have been performed.^6^,^7^ The rationale behind adding radiotherapy to neoadjuvant chemotherapy is to improve locoregional control by sterilizing vessel margins and enhancing the likelihood of a radical (R0) resection, thereby potentially preventing or postponing locoregional recurrence. Indeed, before the era of FOLFIRINOX (5-fluorouracil with leucovorin, irinotecan, and oxaliplatin), several phase 2 and phase 3 studies of neoadjuvant radiotherapy combined with single- or double-agent chemotherapy have consistently shown high R0 resection rates.^8^–^13^

Multidrug regimens including FOLFIRINOX and gemcitabine with nab-paclitaxel have shown superiority to gemcitabine in randomized trials in metastatic and adjuvant settings.^14^–^16^ Based on extrapolation of these results, FOLFIRINOX is commonly used in the neoadjuvant setting in many centers worldwide nowadays. Two patient-level meta-analyses of observational studies in patients with locally advanced pancreatic cancer (LAPC) and BRPC treated with FOLFIRINOX ± radiotherapy indeed showed promising results.^17^,^18^ Due to limited high-level evidence, current guidelines do not draw final conclusions on whether these multidrug regimens should be combined with radiotherapy.^3^–^5^ The role of neoadjuvant radiotherapy in addition to neoadjuvant FOLFIRINOX in patients with (B)RPC remains unclear. Published prospective and retrospective observational studies on this topic are small, precluding definitive conclusions on outcomes.

The aim of this systematic review and meta-analysis was to compare outcomes of (B)RPC patients who received neoadjuvant FOLFIRINOX alone versus FOLFIRINOX with neoadjuvant radiotherapy.

## Methods

### Search Strategy

This systematic review and meta-analysis was performed according to the PRISMA guidelines.^19^ An extensive librarian-led literature search of Embase, MEDLINE (via OvidSP), Web-of-Science, Scopus, Cochrane Central, and Google Scholar was performed on 18 December 2020. The search strategy included the following terms: “FOLFIRINOX,” “folinic acid,” “fluorouracil,” “irinotecan,” “oxaliplatin,” “drug combination,” “pancreatic cancer,” and relevant variants. A full description of the search strategy is outlined in Supplementary Table 1. No restrictions on publication dates were applied.

### Eligibility

Eligible studies reported outcomes for treatment-naïve patients with resectable or borderline resectable pancreatic cancer [(B)RPC] as defined within each study, and who were either treated with neoadjuvant FOLFIRINOX alone (FOLFIRINOX alone group) or with neoadjuvant FOLFIRINOX followed by any type of radiotherapy (FOLFIRINOX with radiotherapy group). To adequately compare the treatment strategies, additional eligibility criteria were applied. Prospective studies were eligible if patients were scheduled to receive either FOLFIRINOX alone or FOLFIRINOX combined with radiotherapy. Retrospective studies were eligible as FOLFIRINOX with radiotherapy study if at least 90% of patients received radiotherapy following FOLFIRINOX and as FOLFIRINOX alone study if less than 10% of patients received additional radiotherapy. Reviews, letters to the editor, case reports, conference abstracts, and articles written in language other than English were excluded.

### Selection Procedure and Data Collection

After removal of duplicates, two authors (Q.J. and I.K.) independently screened the abstracts for eligibility. Full-text assessment was performed for all studies that met the inclusion criteria. Articles were excluded if none of the primary or secondary outcomes were reported or if the same cohort was presented in another study. Discordant judgments were addressed through discussion until consensus was achieved. Data were extracted from the articles separately by the first and second author using a standardized data extraction form.

### Methodological Assessment

Risk of bias was assessed using the Critical Appraisal Skill Program (CASP) appraisal system, which is designed to systematically assess the methodological quality of studies.^20^ Publication bias was assessed using a funnel plot.^21^

### Statistical Analysis

The primary outcome was median OS, as reported by the included articles or extracted from the survival curves. The weighted pooled estimate of median OS was calculated using the formula proposed in a previous meta-analysis, with a study-specific weight function based on the number of patients of interest.^6^ For the primary analysis, the median OS by intention to treat was used (e.g., excluding studies only reporting outcomes for patients who underwent a resection). Furthermore, the pooled weighted median OS in patients who ultimately underwent resection was calculated. For studies reporting the latter outcome from time of resection, the median OS time was increased with the estimated duration of neoadjuvant treatment based on the reported median number of cycles plus 1 month as estimated time between the end of chemotherapy and surgery date. Confidence intervals for median survival estimates were not calculable, and therefore, the range of medians was provided.

Secondary outcomes were progression-free survival (PFS) in patients who underwent resection, resection rate, adjuvant therapy rate, and postoperative outcomes including R0 resection rate (i.e., among patients who underwent resection and among all patients who started neoadjuvant treatment), ypN0 rate, perineural invasion rate, and pathologic complete response rate. For the adjuvant therapy rate, all patients from prospective studies were included in the denominator, since it is likely that this outcome will be known and reported for prospective studies. Patients from retrospective studies were only included in the denominator for the adjuvant therapy rate if this outcome was reported, since the lack of reporting may be due to information bias. Studies only reporting outcomes for patients who ultimately underwent resection were excluded for calculation of the pooled resection rate, yet included for the pooled R0 resection rate and other pathologic outcomes. Random-effects rather than fixed-effects models were used for all pooled analyses to account for potential between-study heterogeneity and *I*^2^ was used as a measure of consistency across studies. Pooled analyses were performed using the meta package for R 3.5.0. All tests were two-sided, and a *p*-value less than .05 was considered statistically significant.

## Results

### Included Studies

**Fig. 1:**
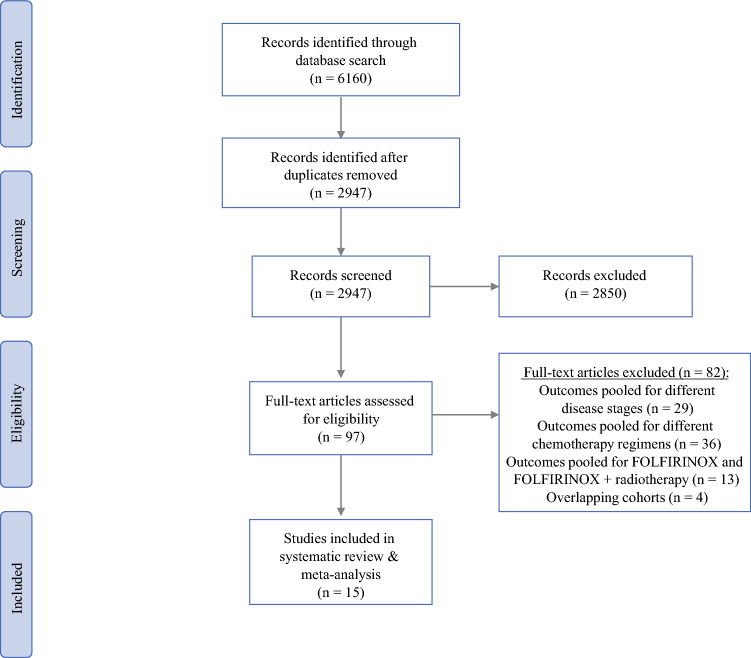
PRISMA flowchart showing selection of articles for systematic review and metaanalysis

The literature search identified 6160 records. After removal of duplicates, 2947 records were screened for eligibility. Based on title and abstract, 97 studies were selected for full-text assessment of which 15 fulfilled all inclusion criteria (Fig. 1). The reason for exclusion based on full-text assessment is outlined in Supplementary Table 3.

Table 1 presents the study characteristics of the 15 included studies. In total, 1081 patients with pancreatic cancer were included, of whom 512 met eligibility criteria based on stage and treatment. Eight studies included 351 patients (68.6%) who received neoadjuvant FOLFIRINOX alone, and 7 other studies included 161 patients (31.4%) who received neoadjuvant FOLFIRINOX followed by radiotherapy. Twelve studies reported outcomes for BRPC patients specifically.^22^–^33^ Three studies also or solely reported outcomes for patients with resectable pancreatic cancer.^34^–^36^ In total, the FOLFIRINOX alone studies included 310 patients (88.3%) with BRPC and 41 patients (11.7%) with resectable pancreatic cancer, whereas all 161 patients (100.0%) in the FOLFIRINOX with radiotherapy studies had BRPC. Four studies included only patients who underwent a resection after neoadjuvant treatment, 25,32,35,36 while the other 11 studies included all patients who started neoadjuvant treatment.

**Table 1:** Study characteristics

Study (reference)	Country	Evidence	Inclusion period	Total ^a^	Total (B)RPC + FFX	Definition resectability	Radiotherapy, No. (%)	Chemotherapy		Radiotherapy			Adjuvant therapy, No. (%)
								Regimen	Neoadj. cycles, median (range)	Type	CRT regimen	Dose, fractions	
*FOLFIRINOX alone*													
Barenboim^22^	Israel	Retrospective	2008–2017	100	23	NCCN	2 (8.7)	FOLFIRINOX	8 (5–14)	–	–	–	16 (70.0)
Dhir^36^	USA	Retrospective	2011–2017	193	73^b^	NCCN	0 (0.0)	FOLFIRINOX	3 (IQR 3–4)	–	–	–	54 (73.9)
Okada^23^	Japan	Prospective	2014–2015	10	10	NCCN	0 (0.0)	mFOLFIRINOX ^e^	6 (4–8)	–	–	–	8 (80.0)
Tinchon^24^	Austria	Prospective	2010–2012	12	10	AHPBA/SSO/SSAT	0 (0.0)	FOLFIRINOX	4 (4–6)	–	–	–	0
De Marsh^34^	USA	Prospective	2013–2015	21	21^c^	NCCN	0 (0.0)	mFOLFIRINOX	4 (NR–4)	–	–	–	15 (71.4)
Kim^35^	USA	Retrospective	2011–2015	26	18^d^	NCCN	0 (0.0)	FOLFIRINOX	9 (4–12)	–	–	–	7 (38.9)
Medrano^25^	France	Retrospective	2011–2018	139	121	NCCN	0 (0.0)	FOLFIRINOX	4 (4–16)	–	–	–	76 (62.8)
Yoo^26^	South Korea	Retrospective	2013–2017	199	75	NCCN	0 (0.0)	(m)FOLFIRINOX	7 (1–41)	–	–	–	NR
Total				700	351		2 (0.6)		5 (3–9)				176 (58.2)
*FOLFIRINOX with radiotherapy*													
Christians^27^	USA	Retrospective	2010–2012	18	18	MCW	18 (100.0)	FOLFIRINOX	4 (3–8)	CRT	Gemcitabine/ capecitabine	50.4 Gy, 28	1 (5.6)
Katz^28^	USA	Prospective	2013–2014	22	22	ALLIANCE	21 (95.5)	mFOLFIRINOX	4 (NR)	CRT	Capecitabine	50.4 Gy, 28	10 (45.5)
Murphy^29^	USA	Prospective	2012–2016	48	48	NR	44 (91.7)	FOLFIRINOX	8 (NR)	CRT	Capecitabine/5-fluorouracil	25.0 Gy, 5 /30.0 Gy, 10 /50.4 Gy, 28 ^g^	5 (10.4)
Shaib^30^	USA	Prospective	NR	13	13	ALLIANCE	12 (92.3)	mFOLFIRINOX	4 (NR)	SBRT	–	36 - 45 Gy, 3	0
Tran^31^	USA	Prospective	2011–2017	25	25	NCCN	19 (76.0)	(m)FOLFIRINOX	6 (NR)	CRT	Gemcitabine	50.0 Gy, 25	0
Bolton^32^	USA	Retrospective	2008–2015	195	31	AHPBA/SSO/SSAT	28 (90.3)^f^	FOLFIRINOX	4 (3–5)	CRT	5-Fluorouracil	NR	NR
Mahaseth^33^	USA	Retrospective	2010–2012	60	4	NR	4 (100.0)	mFOLFIRINOX	NR (2–6)	CRT	Gemcitabine/capecitabine	NR	NR
Total				381	161		146 (90.7)		4 (4–8)				16 (6.0)

*AHPBA/SSO/SSAT* Americas Hepato-Pancreato-Biliary Association/Society of Surgical Oncology/Society for Surgery of the Alimentary Tract, *(B)RPC* resectable or borderline resectable pancreatic cancer, *CRT* chemoradiotherapy, *FFX* FOLFIRINOX, *MCW* Medical College of Wisconsin, *MDACC* MD Anderson Cancer Center, *NCCN* National Comprehensive Cancer Network, *No.* number, *NR* not reported, *Neoadj.* neoadjuvant, *SBRT* stereotactic body radiation therapy

^a^Total patients included in study, ^b^Including upfront resectable pancreatic cancer (*n* = 15), ^c^All upfront resectable pancreatic cancer, ^d^Including upfront resectable pancreatic cancer (*n* = 5), ^e^Modified FOLFIRINOX without bolus 5-fluorouracil and leucovorin, and with reduced dose of irinotecan, ^f^Estimation based on percentage mentioned in manuscript, ^g^Depending on evaluation by multidisciplinary team; short-course chemotherapy with protons or photons for clearly resectable disease, long-course chemoradiotherapy using intensity modulated radiotherapy in case of persistent vascular involvement

### Methodological Assessment

Seven studies were prospective nonrandomized studies, and 8 studies had a retrospective design (Table 1). No randomized controlled trials were identified. Results of the methodological assessment and funnel plot assessing publication bias are shown in the supplementary section. No study was assessed to contain high risk of bias (Suppl. Table 2). Based on the 8 studies reporting the primary outcome, there was no convincing evidence of publication bias, though 2 studies may be considered an outlier (Suppl. Fig. 1). Since there were no randomized studies, confounding by indication cannot be ruled out.

### Chemotherapy Regimens and Radiotherapy

Details of the chemotherapy and radiotherapy regimens are presented in Table 1. FOLFIRINOX was administered in 9 studies, modified FOLFIRINOX (mFOLFIRINOX) in 5 studies, and 2 studies administered both [(m)FOLFIRINOX]. Dose modifications consisted of the exclusion of 5-fluorouracil bolus in all 7 studies, 2 studies decreased the dose of irinotecan,^23^,^37^ and one study also left out leucovorin.^23^ The median number of administered neoadjuvant FOLFIRINOX cycles ranged from three to 9 cycles. Adjuvant therapy was administered to 176 patients (58.2%) in the FOLFIRINOX only group (6 studies) and 16 patients (6.0%) in the FOLFIRINOX with radiotherapy group (3 studies). Additional single-agent chemotherapy as radiosensitizer was administered to 133 patients (82.6%) in the FOLFIRINOX with radiotherapy group (6 studies).

In the FOLFIRINOX with radiotherapy group, 146 patients (90.7%) received radiotherapy following FOLFIRINOX, compared with 2 patients (0.6%) in the FOLFIRINOX alone group. Patients were treated with radiation and concurrent chemotherapy (CRT) in 6 studies, while a dose-escalating stereotactic body radiation therapy (SBRT) scheme was used in one study. Total administered dose ranged from 25.0 to 50.4 Gy.

### Survival Analysis

The pooled median OS for all studies was 22.0 months (range 11.0–37.7 months). By treatment group, the estimated median OS was 21.6 months (range 18.4–34.0 months) in the FOLFIRINOX only group (3 studies) versus 22.4 months (range 11.0–37.7) in the FOLFIRINOX with radiotherapy group (5 studies) (Table 2). In a sensitivity analysis excluding one study in which a dose-escalating SBRT regimen rather than chemoradiotherapy was used, the median OS for the FOLFIRINOX with radiotherapy group (4 studies) was 25.4 months (range 15.8–37.7 months).

**Table 2:** Survival outcomes for (B)RPC patients treated with (m)FOLFIRINOX as first-line treatment with or without additional radiotherapy

Study (reference)	No. of (B)RPC patients	Median FU, months	Baseline for OS and PFS calculations	Median OS, months (95% CI)		Median PFS, months (95% CI)
				All patients	Resected patients	Resected patients
*FOLFIRINOX alone*						
Barenboim^22^	23	17.0	Start treatment	27.9 (NR)	34.3 (NR)	13.7 (NR)
Dhir^36^	73^a^	35.8	Diagnosis	#	38.7 (25.7–50.6)	NR
Okada^23^	10	NR	NR	NR	NR	NR
Tinchon^24^	10	15.4	Start treatment	Not reached	Not reached	Not reached
De Marsh^34^	21^b^	27.7	Start treatment	34 (12.3-57.6)	35.5 (15.0–59.2)	15.2 (10.5–24.1)
Kim^35^	13^c^	41.4^c^	Start treatment	#	34.2 (NR)^c^	19.6 (NR)^c^
Medrano^25^	121	Mean 39	Diagnosis	#	45.0 (NR)	28.0 (NR)
Yoo^26^	75	40.3	Start treatment	18.4 (16.1–20.8)	NR	NR
Estimated median survival (months)	351	33.3		21.6 (range 18.4–34.0)	40.4 (range 34.2–45.0)	22.1 (range 13.7–28.0)
*FOLFIRINOX with radiotherapy*						
Christians^27^	18	22.0	Diagnosis	15.8 (NR)^d^	Not reached	NR
Katz^28^	22	NR	Trial registration	21.7 (15.7–not reached)	23.1 (NR)^d^	At 12 months: 53% (33–86); At 18 months: 40% (19–84)
Murphy^29^	48	18.0	Start treatment	37.7 (19.4–not reached)	Not reached	48.6 (14.4–not reached)
Shaib^30^	13	18.0	Trial registration	11.0 (5.8–not reached)	Not reached (9.3–not reached)	29.6 (5.1–not reached)
Tran^31^	25	NR	Trial registration	24.4 (12.6–40.0)	37.1 (15.4–not reached)	21.6 (8.2–31.7)
Bolton^32^	31	NR	Resection	#	42.5 (NR)^e^	NR
Mahaseth^33^	4	NR	Start treatment	NR	NR	NR
Estimated median survival (months)	161	18.8		22.4 (range 11.0–37.7)	33.5 (range 23.1–42.5)	28.4 (range 18.0–48.6)^f^

*(B)RPC* resectable or borderline resectable pancreatic cancer, *CI* confidence interval, *FU* follow-up, *No.* number, *NR* not reported, *OS* overall survival, *PFS* progression-free survival

^a^Including upfront resectable pancreatic cancer (*n* = 15), ^b^All upfront resectable pancreatic cancer, ^c^Survival outcomes for subgroup of BRPC patients specifically, ^d^Estimation based on Kaplan–Meier results, ^e^Based on Kaplan–Meier results of BRPC patients who received 4 or more cycles of neoadjuvant FOLFIRINOX, with 3 months added for duration of neoadjuvant treatment and time to surgery, ^f^Pooled estimate including a median PFS of 18 months for Katz et al.,^28^ since the median PFS certainly did not exceed 18 months

^#^Study only included patients who underwent resection

**Table 3:** Surgical and pathological outcomes for (B)RPC patients treated with (m)FOLFIRINOX as first-line treatment, with or without additional radiotherapy

Study (Reference)	No. of (B)RPC patients	Resection rates		ypN0, no. (%)^f^	Perineural invasion, No. (%)^f^	Pathological complete response, No. (%)^f^
		Resection, No. (%)	R0 resection, No. (%)^e^			
*FOLFIRINOX alone*						
Barenboim^22^	23	20 (87.0)	20 (100.0)	16 (80.0)^d^	13 (65.0)^d^	3 (15.0)^d^
Dhir^36^	73^a^	#	62 (84.9)	32 (43.8)	57 (78.1)	3 (4.1)
Okada^23^	10	7 (70.0)	5 (71.4)	NR	NR	0
Tinchon^24^	10	8 (80.0)	NR	NR	NR	NR
De Marsh^34^	21^b^	17 (81.0)	16 (94.1)	NR	NR	1 (5.9)
Kim^35^	18^c^	#	17 (94.4)	11 (61.1)^e^	10 (55.6)^e^	0
Medrano^25^	121	#	90 (74.4)	40 (33.1)	98 (81.0)	3 (2.5)
Yoo^26^	75	27 (36.0)	NR	NR	NR	NR
Total	276	79 (71.9)	210 (88.0)	99 (52.5)	178 (75.1)	10 (3.9)
*FOLFIRINOX with radiotherapy*						
Christians^27^	18	12 (66.7)	12 (100.0)	10 (83.3)	NR	0
Katz^28^	22	15 (68.0)	14 (93.3)	10 (66.7)	NR	2 (13.3)
Murphy^29^	48	32 (66.7)	31 (96.9)	20 (62.5)	22 (68.8)	0
Shaib^30^	13	8 (61.5)	8 (100.0)	7 (87.5)	7 (87.5)	0
Tran^31^	25	13 (52.0)	13 (100.0)	6 (46.2)	NR	0
Bolton^32^	31	#	NR	NR	NR	4 (12.9)
Mahaseth^33^	4	2 (50.0)	2 (100.0)	2 (100.0)	NR	NR
Total	161	82 (63.1)	80 (97.6)	55 (67.1)	29 (72.5)	6 (2.9)

*(B)RPC* resectable or borderline resectable pancreatic cancer, *No.* number, *NR* not reported, *ypN0* absence of positive lymph nodes.

^a^Including upfront resectable pancreatic cancer (*n* = 15), ^b^All upfront resectable pancreatic cancer, ^c^Including upfront resectable pancreatic cancer (*n* = 5), ^d^Estimated based on percentage of pooled results for borderline resectable (*n* = 20) and locally advanced pancreatic cancer (*n* = 3), ^e^Estimated based on percentage of pooled results for resectable and borderline resectable (*n* = 18) and locally advanced pancreatic cancer (*n* = 4), ^f^Percentage of patients who underwent a resection

#Studies included only patients who underwent a resection

Eight studies reported the median OS specifically for those patients who underwent a resection after neoadjuvant treatment. For this subgroup, the estimated median OS was 40.4 months (range 34.2–45.0 months) in the FOLFIRINOX alone group (5 studies) versus 33.5 months (range 23.1–42.5 months) in the FOLFIRINOX with radiotherapy group (3 studies). Median OS was not reached in 4 studies.

Median PFS in patients who underwent a resection after neoadjuvant treatment is presented in Table 2. The pooled estimated median PFS was 22.1 months (range 13.7–28.0 months) in the FOLFIRINOX alone group (4 studies) versus 28.4 months (range 18.0–48.6 months) in the FOLFIRINOX with radiotherapy group (4 studies).

### Surgical and Pathological Outcomes

**Fig. 2:**
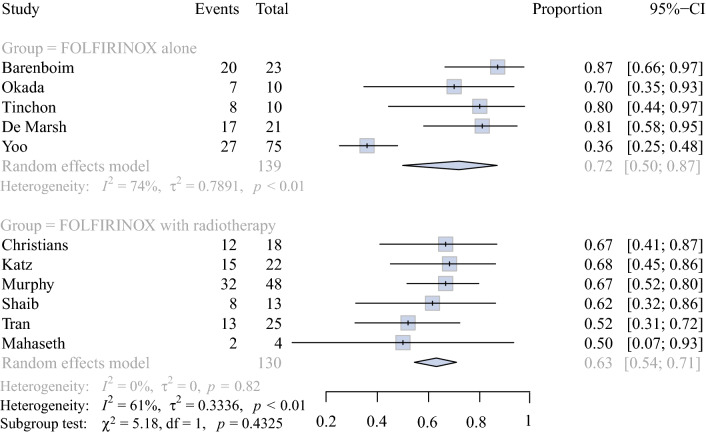
Forest plot showing resection rates in studies with FOLFIRINOX alone versus FOLFIRINOX and radiotherapy (*p* = 0.43). *p-*Value calculated using a two-sided *Q*-test and a random effects model. *CI* confidence interval, *df* degrees of freedom

**Fig. 3:**
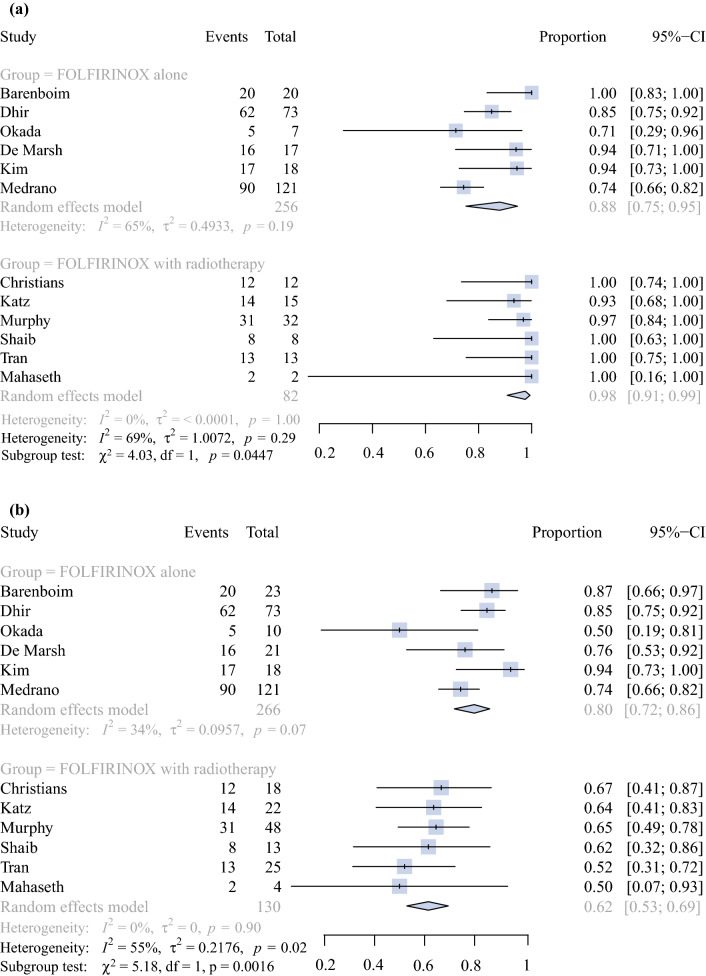
Forest plots showing R0 resection rates. (**a**) Forest plot showing R0 resection rates among patients who underwent a resection in studies with FOLFIRINOX alone versus FOLFIRINOX and radiotherapy (*p* = 0.04). (**b**) Forest plot showing R0 resection rates among all patients starting with neoadjuvant treatment in studies with studies with FOLFIRINOX alone versus FOLFIRINOX and radiotherapy (*p* < 0.01). *p-*Value calculated using a two-sided *Q*-test and a random effects model. *CI* confidence interval, *df* degrees of freedom

Surgical and pathological outcomes are reported in Table 3. Forest plots of pooled resection and R0 resection rates are shown in Figs. 2 and 3, respectively. The pooled resection rate was 71.9% (79/139 patients, 95% CI: 49.9–86.8%) in the FOLFIRINOX alone group (5 studies) versus 63.1% (82/130 patients, 95% CI: 54.5–70.9%) in the FOLFIRINOX with radiotherapy group (6 studies) (*I*^2^ = 61%, *p* = 0.43) (Fig. 2).

Among the patients who underwent a resection, the pooled R0 resection rate was 88.0% (210/256 patients, 95% CI: 75.2–94.7%) in the FOLFIRINOX alone group (6 studies) versus 97.6% (80/82 patients, 95% CI: 90.8–99.4%) in the FOLFIRINOX with radiotherapy group (6 studies) (*I*^2^ = 69%, *p* = 0.045) (Fig. 3a). The pooled R0 resection rate in all patients starting with FOLFIRINOX was 79.9% (210/266 patients, 95% CI: 71.9–86.1%) in the FOLFIRINOX alone group (6 studies) versus 61.5% (80/130 patients, 95% CI: 52.9–69.5%) in the FOLFIRINOX with radiotherapy group (6 studies) (*I*^2^ = 54%, *p* = 0.002) (Fig. 3b).

The pooled ypN0 rate was 52.5% (99/232 patients, 95% CI: 34.0–70.4%) in the FOLFIRINOX alone group (4 studies) versus 67.1% (55/82 patients, 95% CI: 56.2–76.4%) in the FOLFIRINOX with radiotherapy group (6 studies) (*I*^2^ = 73%, *p* = 0.18). The pooled perineural invasion rate was 75.1% (178/232 patients, 95% CI: 63.9–83.7%) in the FOLFIRINOX alone group (4 studies) versus 72.5% (29/40 patients, 95% CI: 56.8–84.1%) in the FOLFIRINOX with radiotherapy group (2 studies) (*I*^2^ = 23%, *p* = 0.77). Pathologic complete response was rare, considering a pooled estimate of 3.9% (10/256 patients, 95% CI: 2.1–7.1%) in the FOLFIRINOX alone group (6 studies) versus 2.9% (6/111 patients, 95% CI: 0.3–21.2%) in the FOLFIRINOX with radiotherapy group (6 studies) (*I*^2^ = 33%, *p* = 0.80).

## Discussion

In this systematic review and meta-analysis including 512 patients with (B)RPC, no difference in survival could be demonstrated between treatment with neoadjuvant FOLFIRINOX with radiotherapy or neoadjuvant FOLFIRINOX alone. The pooled resection rate was also similar, but the pooled R0 resection rate was higher for patients receiving FOLFIRINOX with radiotherapy. These findings support the hypothesis that systemic control remains the most important factor for survival in pancreatic cancer in the era of neoadjuvant FOLFIRINOX. However, these results should be interpreted with caution, since they are based on nonrandomized comparisons of small studies. Considering the small subset of patients with upfront resectable disease, the results of our study are mostly applicable to BRPC patients. A patient-level meta-analysis including 283 BRPC patients who received neoadjuvant FOLFIRINOX found a similar median OS of 22.2 months and a similar resection rate of 67.8%.^18^

The pooled resection rate was comparable between the treatment groups. In contrast, the pooled R0 resection rate among patients undergoing resection, which is most commonly reported in the literature, was superior for the FOLFIRINOX with radiotherapy group. This is consistent with a large retrospective multicentric cohort study from France including BRPC and LAPC patients who underwent a resection after induction FOLFIRINOX combined with chemoradiotherapy (*n* = 102) or FOLFIRINOX alone (*n* = 101). This cohort showed higher R0 (89% vs. 76%, *p* = 0.017) and ypN0 (77% vs. 49%, *p* < 0.001) resection rates in patients who received both FOLFIRINOX and chemoradiotherapy. In addition, patients with additional chemoradiotherapy had significantly longer OS (median OS: 57.8 vs. 35.5 months; *p* = 0.007), which could not be demonstrated in the current meta-analysis.^38^ This may be explained by the inclusion of LAPC patients in the French study.

Focusing on chemotherapy regimens other than FOLFIRINOX with or without radiotherapy, a large Japanese multicentric cohort study included a prospensity-matched analysis of 376 patients with BRPC who received chemotherapy with radiotherapy (mostly gemcitabine- or S1-based chemoradiotherapy) or neoadjuvant chemotherapy alone (mostly gemcitabine + S1). This study showed a higher ypN0 rate (62.2% vs. 34.0%; *p* < 0.001) and lower locoregional recurrence rate (20.4% vs. 44.6%; *p* = 0.002) in the chemotherapy with radiotherapy group, yet no difference in R0 resection rate (87.2% vs. 84.1%, *p* = 0.50) and survival (median OS: 22.5 vs. 29.2 months; *p* = 0.130) could be demonstrated.^39^

No difference in pathological complete response rate could be demonstrated. However, a clinically relevant impact of radiotherapy after FOLFIRINOX on pathologic response cannot be ruled out because of the small number of patients. Two recent retrospective studies found a pathologic complete response rate ranging from 6.8 to 16.3% after systemic chemotherapy and radiotherapy.^40^,^41^ A large study from the National Cancer Database showed that preoperative radiation was independently associated with a pathologic complete response on multivariable analysis.^42^ However, it has not been shown that complete response for a few patients translates into an improvement of survival for all patients who receive neoadjuvant radiation.

Patients in the FOLFIRINOX alone studies have clearly received more adjuvant therapy as compared with patients in the FOLFIRINOX with radiotherapy studies. On the other hand, additional single-agent chemotherapy was used as radiosensitizer in 6 out of the 7 FOLFIRINOX with radiotherapy studies. Since both the neoadjuvant chemoradiotherapy and adjuvant therapy mostly included single-agent chemotherapy regimens, the total systemic treatment may have been comparable in the 2 groups, yet this remains uncertain.

SBRT is a new development in the field of radiotherapy.^43^ By applying image guidance, the tumor can be followed during the radiation (tracking), or radiation can be interrupted when the tumor moves out of the beam (gating). This allows high doses of radiation in a very short period of time with less toxicity than conventional chemoradiotherapy. Several systematic reviews and large epidemiological studies found good results in LAPC.^44^–^46^ Moreover, a recent study in the National Cancer Data Base (NCDB) of over 2000 patients with resected upfront resectable pancreatic cancer who received neoadjuvant multiagent chemotherapy without radiotherapy (*n* = 1355), with conventional radiotherapy (*n* = 552), or with SBRT (*n* = 175), showed superior outcomes for the patients receiving SBRT.^47^ In the propensity-matched analysis, SBRT was associated with a significantly better survival than chemotherapy alone (HR 0.65, 95% CI: 0.47–0.90, *p* = 0.01) and chemotherapy plus conventional radiotherapy (HR 0.53, 95% CI: 0.37–0.76, *p* = 0.001). Furthermore, SBRT was associated with a better R0 resection rate (chemotherapy alone 81% vs. chemotherapy + conventional radiotherapy 86% vs. chemotherapy + SBRT 91%; *p* = 0.0001) and pathologic complete response rate (respectively 2.2% vs. 4.9% vs. 6.1%; *p* = 0.0002).^47^ In line with the current study, this suggests that future randomized studies of neoadjuvant treatment should focus on modern, multiagent chemotherapy in combination with SBRT rather than conventional radiotherapy.

Another new development in the field of radiotherapy for pancreatic cancer is combining radiotherapy with immunotherapeutic agents.^48^,^49^ Both in vitro and in vivo studies have shown that radiotherapy may act as an “in situ vaccine” by increasing the expression of cell surface receptors such as major histocompatibility complex class I (MHC-I) and by increasing tumor antigen presentation.^50^–^52^ However, due to the immune suppressive tumor microenvironment in pancreatic cancer, the antitumor immune response induced by radiotherapy alone may not be sufficient.^53^ When combined, the increased release of tumor-specific antigens by radiotherapy may enhance the efficacy of immotherapeutic drugs, potentially resulting in a robust and targeted antitumor immune response.^48^,^54^

Four ongoing randomized controlled trials may provide better insights in the individual contributions of systemic chemotherapy and radiotherapy for BRPC patients.^55^–^57^ In the ALLIANCE trial A021501, 134 BRPC patients are randomized to neoadjuvant mFOLFIRINOX (8 cycles) or neoadjuvant mFOLFIRINOX (7 cycles) plus SBRT, with surgery and adjuvant FOLFOX in both arms.^55^ In the French PANDAS-PRODIGE 44 trial (NCT02676349), 90 BRPC patients are randomized to neoadjuvant mFOLFIRINOX (8 cycles) or neoadjuvant mFOLFIRINOX (8 cycles) with subsequent capecitabine-based chemoradiotherapy, followed by surgery and adjuvant gemcitabine or 5-FU in both arms. Results of these 2 studies are expected in 2021. The Chinese BRPCNCC-1 trial is a three-arm trial that randomizes 150 BRPC patients to neoadjuvant gemcitabine plus nab-paclitaxel alone, neoadjuvant gemcitabine plus nab-paclitaxel with SBRT, or neoadjuvant S1 plus nab-paclitaxel with SBRT, with expected results in 2022.^56^ Finally, the Dutch PREOPANC-2 trial has completed accrual of 368 (B)RPC patients who were randomized to total neoadjuvant FOLFIRINOX (8 cycles) or neoadjuvant gemcitabine-based chemoradiotherapy and adjuvant gemcitabine, with results expected in 2022.^57^

Some limitations should be taken into account when interpreting the results of our study. First, no randomized trial was included that directly compared FOLFIRINOX with or without radiotherapy. Half of the studies were retrospective studies with potential confounding by indication and information bias. Furthermore, many studies included only small numbers of patients with (B)RPC. Together, these factors have limited the quality of the included studies. Second, our primary endpoint was the estimated median survival time, whereby studies were weighted based on the number of study participants. This weighted estimate of median OS is an imperfect analytical method but a conventional meta-analytical method in the absence of hazard ratios or patient-level data. Third, only one study focused primarily on the addition of radiotherapy to FOLFIRINOX in a dose-finding phase 1 design. This was the only study concerning SBRT. All other studies included conventional chemoradiotherapy, which, as suggested earlier, may not be ideal in this setting. Fourth, heterogeneity across the included studies might have influenced the results, with differences in neoadjuvant FOLFIRINOX treatment (e.g., number of cycles and dose modifications), radiotherapy treatment (e.g., doses, fractions, and concurrent chemotherapy), and different definitions for (B)RPC. This heterogeneity was anticipated by using random effects for all pooled analyses. Last, not all endpoints were reported in several studies, resulting in less precise and potentially biased estimates. Despite these unavoidable limitations, considering the available evidence, the results of the present meta-analysis currently provide the best available comparison of FOLFIRINOX with or without additional radiotherapy in patients with (B)RPC.

In conclusion, radiotherapy following neoadjuvant FOLFIRINOX was associated with an improved R0 resection rate as compared with neoadjuvant FOLFIRINOX alone, but a difference in survival could not be demonstrated. Randomized trials are needed to determine the added value of radiotherapy following neoadjuvant FOLFIRINOX in patients with (B)PRC.

## Supplementary Information

Below is the link to the electronic supplementary material.

Supplementary file1 (PDF 271 kb)
