# Supplementary material for: Added Value of Radiotherapy Following Neoadjuvant FOLFIRINOX for Resectable and Borderline Resectable Pancreatic Cancer: A Systematic Review and Meta-Analysis
Source: Ann Surg Oncol. 2021 Jun 17;28(13):8297–308. doi: 10.1245/s10434-021-10276-8 (PMC8591030; doi:10.1245/s10434-021-10276-8)

SUPPLEMENTARIES FOR: ''The added value of combining radiotherapy with neoadjuvant FOLFIRINOX for resectable and borderline resectable pancreatic cancer: a systematic review and meta-analysis.''

Supplementary Table 1. Search strategy and results.

**Database**	**Search query**	**Number of records**	**After deduplication**
Embase.com	(('folinic acid'/exp AND fluorouracil/exp AND irinotecan/exp AND oxaliplatin/exp AND 'drug combination'/exp AND ('pancreas cancer'/de OR 'pancreas tumor'/de OR 'pancreas adenoma'/de OR 'pancreas adenocarcinoma'/de OR 'pancreas carcinoma'/de OR 'pancreas islet cell carcinoma'/de OR (pancrea* NEAR/3 (cancer* OR neoplas* OR tumo* OR adenocarcinom* OR carcinom* OR adenom*)):ab,ti)) OR (Folfirinox):ab,ti)	2314	2143
Medline (Ovid)	((Leucovorin/ AND fluorouracil/ AND irinotecan.mp. AND oxaliplatin.mp. AND Drug Combinations/ AND (exp Pancreatic Neoplasms/ OR (pancrea* ADJ3 (cancer* OR neoplas* OR tumo* OR adenocarcinom* OR carcinom* OR adenom*)).ab,ti.)) OR (Folfirinox).ab,ti.)	815	48
Web of Science	(TI=(neoadjuvant*) OR AB=(neoadjuvant*)) AND (TI=((pancrea*) NEAR/5 (cancer* OR carcinoma* OR adenocarcinoma* OR neoplas* OR tumor* OR tumour*)) OR AB=((pancrea*) NEAR/5 (cancer* OR carcinoma* OR adenocarcinoma* OR neoplas* OR tumor* OR tumour*))) AND (TI=(random* OR factorial* OR crossover* OR (cross NEAR/1 over*) OR placebo* OR ((doubl* OR singl*) NEAR/1 blind*) OR assign* OR allocat* OR volunteer* OR trial OR groups) OR AB=(random* OR factorial* OR crossover* OR (cross NEAR/1 over*) OR placebo* OR ((doubl* OR singl*) NEAR/1 blind*) OR assign* OR allocat* OR volunteer* OR trial OR groups)) AND LA=English	1587	527
Scopus	TITLE-ABS-KEY(Folfirinox)	923	55
Cochrane Central	(Folfirinox):ab,ti	321	111
Google Scholar	Folfirinox	200	63
**Total**		**6160**	**2947**

Supplementary Table 2. Methodological quality assessment according to Critical Appraisal Skill Program (CASP).

Study	Clear question	Recruitment	Accurately measured exposure	Accurately measured outcome	Confounding factors identified	Confounding factors accounted	Follow-up complete enough	Follow-up long enough	Precise statistical results presented	Do you believe results	Ability to generalize results	In accordance with existing evidence	Implications of study in practice
**Barenboim**	Yes	Yes	Yes	Yes	No	No	Yes	Yes	Yes	Yes	Yes	Yes	No
**Dhir**	Yes	Yes	Yes	Yes	Yes	Yes	Yes	Yes	Yes	Yes	No	Yes	No
**Okada**	Yes	Yes	Yes	Yes	No	No	Yes	Yes	Yes	Yes	Yes	Yes	No
**Tinchon**	Yes	Yes	No	Yes	No	No	Yes	No	No	Yes	Yes	Yes	No
**De Marsh**	Yes	Yes	Yes	Yes	No	No	Yes	Yes	Yes	Yes	Yes	Yes	Yes
**Kim**	Yes	Yes	Yes	Yes	Yes	No	Yes	Yes	Yes	Yes	No	No	No
**Medrano**	Yes	Yes	Yes	Yes	Yes	Yes	Yes	Yes	Yes	Yes	No	No	Yes
**Yoo**	Yes	Yes	Yes	Yes	Yes	Yes	Yes	Yes	Yes	Yes	Yes	Yes	Yes
**Christians**	Yes	Yes	Yes	Yes	No	No	Yes	No	Yes	Yes	Yes	Yes	No
**Katz**	Yes	Yes	Yes	Yes	Yes	No	Yes	Yes	Yes	Yes	Yes	Yes	Yes
**Murphy**	Yes	Yes	Yes	Yes	Yes	No	Yes	Yes	Yes	Yes	Yes	Yes	Yes
**Shaib**	Yes	Yes	Yes	Yes	Yes	No	Yes	Yes	Yes	Yes	Yes	Yes	Yes
**Tran**	Yes	Yes	Yes	Yes	Yes	No	Yes	Yes	Yes	Yes	Yes	Yes	Yes
**Bolton**	Yes	Yes	Yes	Yes	Yes	Yes	Yes	Yes	Yes	Yes	No	Yes	No
**Mahaseth**	Yes	Yes	Yes	Yes	No	No	Yes	No	Yes	Yes	No	Yes	No

Supplementary Table 3: References excluded studies after full text assessment, n=82

**Outcomes pooled for different disease stages, n=29**
1. Harrison JM, Wo JY, Ferrone CR, Horick NK, Keane FK, Qadan M, et al. Intraoperative Radiation Therapy (IORT) for Borderline Resectable and Locally Advanced Pancreatic Ductal Adenocarcinoma (BR/LA PDAC) in the Era of Modern Neoadjuvant Treatment: Short-Term and Long-Term Outcomes. Ann Surg Oncol. 2019.<br>2. Heger U, Sun H, Hinz U, Klaiber U, Tanaka M, Liu B, et al. Induction chemotherapy in pancreatic cancer: CA 19-9 may predict resectability and survival. HPB. 2019.<br>3. Hosein PJ, Macintyre J, Kawamura C, Maldonado JC, Ernani V, Loaiza-Bonilla A, et al. A retrospective study of neoadjuvant FOLFIRINOX in unresectable or borderline-resectable locally advanced pancreatic adenocarcinoma. BMC Cancer. 2012;12.<br>4. Kordes M, Yu JR, Malgerud O, Liljefors MG, Lohr JM. Survival Benefits of Chemotherapy for Patients with Advanced Pancreatic Cancer in A Clinical Real-World Cohort. Cancers. 2019;11(9).<br>5. Nywening TM, Wang-Gillam A, Sanford DE. Phase 1b study targeting tumour associated macrophages with CCR2 inhibition plus FOLFIRINOX in locally advanced and borderline resectable pancreatic …: ncbi.nlm.nih.gov; 2016.<br>6. Pietrasz D, Marthey L, Wagner M, Blanc JF. Pathologic major response after FOLFIRINOX is prognostic for patients secondary resected for borderline or locally advanced pancreatic adenocarcinoma: an …: Springer; 2015.<br>7. Rangelova E, Wefer A, Persson S, Valente R, Tanaka K, Orsini N, et al. Surgery Improves Survival After Neoadjuvant Therapy for Borderline and Locally Advanced Pancreatic Cancer: A Single Institution Experience. Ann Surg. 2019.<br>8. Truty MJ, Kendrick ML, Nagorney DM, Smoot RL, Cleary SP, Graham RP, et al. Factors Predicting Response, Perioperative Outcomes, and Survival Following Total Neoadjuvant Therapy for Borderline/Locally Advanced Pancreatic Cancer. Ann Surg. 2019.<br>9. Badiyan SN, Olsen JR, Lee AY, Yano M, Menias CO, Khwaja S, et al. Induction chemotherapy followed by concurrent full-dose gemcitabine and intensity-modulated radiation therapy for borderline resectable and locally advanced pancreatic adenocarcinoma. Am J Clin Oncol Cancer Clin Trials. 2016;39(1):1-7.<br>10. He J, Blair AB, Groot VP, Javed AA, Burkhart RA, Gemenetzis G, et al. Is a Pathological Complete Response Following Neoadjuvant Chemoradiation Associated with Prolonged Survival in Patients with Pancreatic Cancer? Ann Surg. 2018;268(1):1-8.<br>11. Chapman BC, Gleisner A, Rigg D, Messersmith W, Paniccia A, Meguid C, et al. Perioperative and Survival Outcomes Following Neoadjuvant FOLFIRINOX versus Gemcitabine Abraxane in Patients with Pancreatic Adenocarcinoma. JOP. 2018;19(2):75-85.<br>12. Pietrasz D, Turrini O, Vendrely V, Simon JM, Hentic O, Coriat R, et al. How Does Chemoradiotherapy Following Induction FOLFIRINOX Improve the Results in Resected Borderline or Locally Advanced Pancreatic Adenocarcinoma? An AGEO-FRENCH Multicentric Cohort. Ann Surg Oncol. 2019;26(1):109-17.<br>13. Macedo FI, Ryon E, Maithel SK, Lee RM, Kooby DA, Fields RC, et al. Survival Outcomes Associated with Clinical and Pathological Response Following Neoadjuvant FOLFIRINOX or Gemcitabine/Nab-Paclitaxel Chemotherapy in Resected Pancreatic Cancer. Ann Surg. 2019;270(3):400-13.

14. Kang H, Jo JH, Lee HS, Chung MJ, Bang S, Park SW, et al. Comparison of efficacy and safety between standard-dose and modified-dose FOLFIRINOX as a first-line treatment of pancreatic cancer. World J Gastrointest Oncology. 2018;10(11):421-30.
15. Peddi PF, Lubner S, McWilliams R, Tan BR, Picus J, Sorscher SM, et al. Multi-institutional experience with FOLFIRINOX in pancreatic adenocarcinoma. J Pancreas. 2012;13(5):497-501.
16. Wo JY, Niemierko A, Ryan DP, Blaszkowsky LS, Clark JW, Kwak EL, et al. Tolerability and Long-term Outcomes of Dose-Painted Neoadjuvant Chemoradiation to Regions of Vessel Involvement in Borderline or Locally Advanced Pancreatic Cancer. Am J Clin Oncol-Cancer Clin Trials. 2018;41(7):656-61.
17. Kourie H, Auclin E, Cunha AS, Gaujoux S, Bruzzi M, Sauvanet A, et al. Characteristic and outcomes of patients with pathologic complete response after preoperative treatment in borderline and locally advanced pancreatic adenocarcinoma: An AGEO multicentric retrospective cohort. Clin Res Hepatol Gastroenterol. 2019;43(6):663-8.
18. Michelakos T, Pergolini I, Castillo CFD, Honselmann KC, Cai L, Deshpande V, et al. Predictors of Resectability and Survival in Patients With Borderline and Locally Advanced Pancreatic Cancer who Underwent Neoadjuvant Treatment With FOLFIRINOX. Ann Surg. 2019;269(4):733-40.
19. Hashemi-Sadraei N, Gbolahan OB, Salfity H, O'Neil B, House MG, Shahda S. Clinical Characteristics of Patients Experiencing Pathologic Complete Response Following Neoadjuvant Therapy for Borderline Resectable/Locally Advanced Pancreatic Adenocarcinoma. Am J Clin Oncol Cancer Clin Trials. 2018;41(10):982-5.
20. Mancini BR, Stein S, Lloyd S, Rutter CE, James E, Chang BW, et al. Chemoradiation after FOLFIRINOX for borderline resectable or locally advanced pancreatic cancer. J Gastrointest Oncol. 2018;9(6):982-8.
21. Pouypoudat C, Buscail E, Cossin S, Cassinotto C, Terrebonne E, Blanc JF, et al. FOLFIRINOX-based neoadjuvant chemoradiotherapy for borderline and locally advanced pancreatic cancer: A pilot study from a tertiary centre. Dig Liver Dis. 2019;51(7):1043-9.
22. Blazer M, Wu C, Goldberg RM, Phillips G, Schmidt C, Muscarella P, et al. Neoadjuvant Modified (m) FOLFIRINOX for Locally Advanced Unresectable (LAPC) and Borderline Resectable (BRPC) Adenocarcinoma of the Pancreas. Ann Surg Oncol. 2015;22(4):1153-9.
23. Choi YH, Lee SH, You MS, Shin BS, Paik WH, Ryu JK, Kim YT, Kwon W, Jang JY, Kim SW. Prognostic Factors for Patients with Borderline Resectable or Locally Advanced Pancreatic Cancer Receiving Neoadjuvant FOLFIRINOX. Gut Liver. 2020 Apr 2. doi: 10.5009/gnl19182. Epub ahead of print. PMID: 32235008.
24. Hue JJ, Sugumar K, Bingmer K, Ammori JB, Winter JM, Hardacre JM. Neoadjuvant chemoradiation may be associated with improved pathologic response in pancreatic cancer. Am J Surg. 2020 Nov 19:S0002-9610(20)30758-3. doi: 10.1016/j.amjsurg.2020.11.035. Epub ahead of print. PMID: 33234234.
25. Kayahan SatiŞ N, Karaca M, SatiŞ H, Yapar D, Özet A, Özet A. Folfirinox versus gemcitabine-cisplatin combination as first-line therapy in treatment of pancreaticobiliary cancer. Turk J Med Sci. 2020 Dec 14. doi: 10.3906/sag-2009-115. Epub ahead of print. PMID: 33315355.
26. Xie L, Xia L, Klaiber U, Sachsenmaier M, Hinz U, Bergmann F, Strobel O, Büchler MW, Neoptolemos JP, Fortunato F, Hackert T. Effects of neoadjuvant FOLFIRINOX and gemcitabine-based chemotherapy on cancer cell survival and death in patients with pancreatic ductal adenocarcinoma. Oncotarget. 2019 Dec 31;10(68):7276-7287. doi: 10.18632/oncotarget.27399. PMID: 31921387; PMCID: PMC6944451.

27. Cecchini M, Miccio JA, Pahade J, Lacy J, Salem RR, Johnson SB, Blakaj A, Stein S, Kortmansky JS, Johung KL. A Single-Institution Experience of Induction 5-Fluorouracil, Leucovorin, Irinotecan, and Oxaliplatin Followed by Surgery Versus Consolidative Radiation for Borderline and Locally Advanced Unresectable Pancreatic Cancer. Pancreas. 2020 Aug;49(7):904-911. doi: 10.1097/MPA.0000000000001592. PMID: 32658074.
28. Weniger M, Moir J, Damm M, Maggino L, Kordes M, Rosendahl J, Ceyhan GO, Schorn S; RESPECT-study group. Respect - A multicenter retrospective study on preoperative chemotherapy in locally advanced and borderline resectable pancreatic cancer. Pancreatology. 2020 Sep;20(6):1131-1138. doi: 10.1016/j.pan.2020.06.012. Epub 2020 Jul 9. PMID: 32739267.
29. Wolfe AR, Prabhakar D, Yildiz VO, Cloyd JM, Dillhoff M, Abushahin L, Alexandra Diaz D, Miller ED, Chen W, Frankel WL, Noonan A, Williams TM. Neoadjuvant-modified FOLFIRINOX vs nab-paclitaxel plus gemcitabine for borderline resectable or locally advanced pancreatic cancer patients who achieved surgical resection. Cancer Med. 2020 Jul;9(13):4711-4723. doi: 10.1002/cam4.3075. Epub 2020 May 16. PMID: 32415696; PMCID: PMC7333854.

Outcomes pooled for different chemotherapy regimens, n=36

30. Lof S, Korrel M, van Hilst J, Alseidi A, Balzano G, Boggi U, et al. Impact of Neoadjuvant Therapy in Resected Pancreatic Ductal Adenocarcinoma of the Pancreatic Body or Tail on Surgical and Oncological Outcome: A Propensity-Score Matched Multicenter Study. Ann Surg Oncol. 2019.
31. Tsiotos GG, Ballian N, Michelakos T, Milas F, Ziogou P, Papaioannou D, et al. Portal-Mesenteric Vein Resection in Borderline Pancreatic Cancer; 33 Month-Survival in Patients with Good Performance Status. J Pancreatic Cancer. 2019;5(1):43-50.
32. Cloyd JM, Katz MHG, Prakash L, Varadhachary GR, Wolff RA, Shroff RT, et al. Preoperative Therapy and Pancreatoduodenectomy for Pancreatic Ductal Adenocarcinoma: a 25-Year Single-Institution Experience. J Gastrointest Surg. 2017;21(1):164-74.
33. Cloyd JM, Chen HC, Wang XM, Tzeng CWD, Kim MP, Aloia TA, et al. Chemotherapy Versus Chemoradiation as Preoperative Therapy for Resectable Pancreatic Ductal Adenocarcinoma A Propensity Score Adjusted Analysis. Pancreas. 2019;48(2):216-22.
34. Barnes CA, Chavez MI, Tsai S, Aldakkak M, George B, Ritch PS, et al. Survival of patients with borderline resectable pancreatic cancer who received neoadjuvant therapy and surgery. Surgery. 2019;166(3):277-85.
35. Kim SS, Ko AH, Nakakura EK, Wang ZJ, Corvera CU, Harris HW, et al. Comparison of Tumor Regression Grading of Residual Pancreatic Ductal Adenocarcinoma Following Neoadjuvant Chemotherapy Without Radiation Would Fewer Tier-Stratification Be Favorable Toward Standardization? Am J Surg Pathol. 2019;43(3):334-40.
36. Franko J, Hsu HW, Thirunavukarasu P, Frankova D, Goldman CD. Chemotherapy and radiation components of neoadjuvant treatment of pancreatic head adenocarcinoma: Impact on perioperative mortality and long-term survival. Ejso. 2017;43(2):351-7.
37. Peng JS, Wey J, Chalikonda S, Allende DS, Walsh RM, Morris-Stiff G. Pathologic tumor response to neoadjuvant therapy in borderline resectable pancreatic cancer. Hepatob Pancreatic Dis Int. 2019;18(4):373-8.
38. Cools KS, Sanoff HK, Kim HJ, Yeh JJ, Stitzenberg KB. Impact of neoadjuvant therapy on postoperative outcomes after pancreaticoduodenectomy. J Surg Oncol. 2018;118(3):455-62.
39. Lewis S, Sastri SC, Arya S, Mehta S, Patil P, Shrivastava S, et al. Dose escalated concurrent chemo-radiation in borderline resectable and locally advanced pancreatic cancers with tomotherapy based intensity modulated radiotherapy: a phase II study. J Gastrointest Oncol. 2019;10(3):474-82.
40. Blaszak M, El-Masri M, Hirmiz K, Mathews J, Omar A, Elfiki T, et al. Survival of patients with pancreatic cancer treated with varied modalities: A single-centre study. Mol Clin Oncol. 2017;6(4):583-8.

41. Keane FK, Wo JY, Ferrone CR, Clark JW, Blaszkowsky LS, Allen JN, et al. Intraoperative Radiotherapy in the Era of Intensive Neoadjuvant Chemotherapy and Chemoradiotherapy for Pancreatic Adenocarcinoma. Am J Clin Oncol Cancer Clin Trials. 2018;41(6):607-12.
42. Marchegiani G, Andrianello S, Nessi C, Sandini M, Maggino L, Malleo G, et al. Neoadjuvant Therapy Versus Upfront Resection for Pancreatic Cancer: The Actual Spectrum and Clinical Burden of Postoperative Complications. Ann Surg Oncol. 2018;25(3):626-37.
43. Berriochoa CA, Abdel-Wahab M, Leyrer CM, Khorana A, Matthew Walsh R, Kumar AMS. Neoadjuvant chemoradiation for non-metastatic pancreatic cancer increases margin-negative and node-negative rates at resection. Journal of Digestive Diseases. 2017;18(11):642-9.
44. Nurmi A, Mustonen H, Parviainen H, Peltola K, Haglund C, Seppänen H. Neoadjuvant therapy offers longer survival than upfront surgery for poorly differentiated and higher stage pancreatic cancer. Acta Oncol. 2018;57(6):799-806.
45. Maggino L, Malleo G, Marchegiani G, Viviani E, Nessi C, Ciprani D, et al. Outcomes of Primary Chemotherapy for Borderline Resectable and Locally Advanced Pancreatic Ductal Adenocarcinoma. JAMA Surg. 2019;154(10):932-42.
46. Sugimoto M, Takahashi N, Farnell MB, Smyrk TC, Truty MJ, Nagorney DM, et al. Survival benefit of neoadjuvant therapy in patients with non-metastatic pancreatic ductal adenocarcinoma: A propensity matching and intention-to-treat analysis. J Surg Oncol. 2019;120(6):976-84.
47. Mellon EA, Hoffe SE, Springett GM, Frakes JM, Strom TJ, Hodul PJ, et al. Long-term outcomes of induction chemotherapy and neoadjuvant stereotactic body radiotherapy for borderline resectable and locally advanced pancreatic adenocarcinoma. Acta Oncol. 2015;54(7):979-85.
48. Cloyd JM, Wang HM, Egger ME, Tzeng CWD, Prakash LR, Maitra A, et al. Association of Clinical Factors With a Major Pathologic Response Following Preoperative Therapy for Pancreatic Ductal Adenocarcinoma. JAMA Surg. 2017;152(11):1048-56.
49. Blair AB, Rosati LM, Rezaee N, Gemenetzis G, Zheng L, Hruban RH, et al. Postoperative complications after resection of borderline resectable and locally advanced pancreatic cancer: The impact of neoadjuvant chemotherapy with conventional radiation or stereotactic body radiation therapy. Surgery. 2018;163(5):1090-6.
50. Shrestha B, Sun YF, Faisal F, Kim V, Soares K, Blair A, et al. Long-term survival benefit of upfront chemotherapy in patients with newly diagnosed borderline resectable pancreatic cancer. Cancer Med. 2017;6(7):1552-62.
51. Groot VP, Blair AB, Gemenetzis G, Ding D, Burkhart RA, Yu J, et al. Recurrence after neoadjuvant therapy and resection of borderline resectable and locally advanced pancreatic cancer. Ejso. 2019;45(9):1674-83.
52. Cloyd JM, Crane CH, Koay EJ, Das P, Krishnan S, Prakash L, et al. Impact of hypofractionated and standard fractionated chemoradiation before pancreatoduodenectomy for pancreatic ductal adenocarcinoma. Cancer. 2016;122(17):2671-9.
53. Ahmad SA, Duong M, Sohal DPS, Gandhi NS, Beg MS, Wang-Gillam A, Wade JL 3rd, Chiorean EG, Guthrie KA, Lowy AM, Philip PA, Hochster HS. Surgical Outcome Results From SWOG S1505: A Randomized Clinical Trial of mFOLFIRINOX Versus Gemcitabine/Nab-paclitaxel for Perioperative Treatment of Resectable Pancreatic Ductal Adenocarcinoma. Ann Surg. 2020 Jul 24. doi: 10.1097/SLA.0000000000004155. Epub ahead of print. PMID: 32740235.
54. Kim RY, Christians KK, Aldakkak M, Clarke CN, George B, Kamgar M, Khan AH, Kulkarni N, Hall WA, Erickson BA, Evans DB, Tsai S. Total Neoadjuvant Therapy for Operable Pancreatic Cancer. Ann Surg Oncol. 2020 Sep 30. doi: 10.1245/s10434-020-09149-3. Epub ahead of print. PMID: 33000372.

55. Kubo K, Wadasaki K, Komichi D, Sasaki T, Yamada H, Matsugu Y, Itamoto T, Doi M, Shinozaki K. A single institution experience of the treatment of pancreatic ductal carcinoma: The demand and the role of radiation therapy. PLoS One. 2019 Dec 30;14(12):e0227305. doi: 10.1371/journal.pone.0227305. PMID: 31887205; PMCID: PMC6936878.
56. Ocuin LM, Hardacre JM, Ammori JB, Rothermel LD, Mohamed A, Selfridge JE, Bajor D, Winter JM. Neoadjuvant chemotherapy is associated with improved survival in patients with left-sided pancreatic adenocarcinoma. J Surg Oncol. 2020 Aug 25. doi: 10.1002/jso.26196. Epub ahead of print. PMID: 32844445.
57. Trinh KV, Fischer DA, Gardner TB, Smith KD. Outcomes of Neoadjuvant Chemoradiation With and Without Systemic Chemotherapy in Resectable and Borderline Resectable Pancreatic Adenocarcinoma. Front Oncol. 2020 Sep 16;10:1461. doi: 10.3389/fonc.2020.01461. PMID: 33042792; PMCID: PMC7525017.
58. Yoo C, Shin SH, Kim KP, Jeong JH, Chang HM, Kang JH, Lee SS, Park DH, Song TJ, Seo DW, Lee SK, Kim MH, Park JH, Hwang DW, Song KB, Lee JH, Ryoo BY, Kim SC. Clinical Outcomes of Conversion Surgery after Neoadjuvant Chemotherapy in Patients with Borderline Resectable and Locally Advanced Unresectable Pancreatic Cancer: A Single-Center, Retrospective Analysis. Cancers (Basel). 2019 Feb 26;11(3):278. doi: 10.3390/cancers11030278. PMID: 30813624; PMCID: PMC6468804.
59. Han S, Choi SH, Choi DW, Heo JS, Han IW, Park DJ, Ryu Y. Neoadjuvant therapy versus upfront surgery for borderline-resectable pancreatic cancer. Minerva Chir. 2020 Feb;75(1):15-24. doi: 10.23736/S0026-4733.19.07958-6. Epub 2019 May 20. PMID: 31115240.
60. Weniger M, Moir J, Damm M, Maggino L, Kordes M, Rosendahl J, Ceyhan GO, Schorn S; RESPECT-study group. Neoadjuvant therapy in elderly patients receiving FOLFIRINOX or gemcitabine/nab-paclitaxel for borderline resectable or locally advanced pancreatic cancer is feasible and lead to a similar oncological outcome compared to non-aged patients - Results of the RESPECT-Study. Surg Oncol. 2020 Dec;35:285-297. doi: 10.1016/j.suronc.2020.08.031. Epub 2020 Sep 7. PMID: 32949968.
61. Kizy S, Altman AM, Wirth KM, Marmor S, Hui JYC, Tuttle TM, Lou E, Amin K, Denbo JW, Jensen EH. Systemic therapy without radiation may be appropriate as neoadjuvant therapy for localized pancreas cancer. Hepatobiliary Surg Nutr. 2020 Jun;9(3):296-303. doi: 10.21037/hbsn.2019.04.17. PMID: 32509815; PMCID: PMC7262615.
62. Borhani AA, Dewan R, Furlan A, Seiser N, Zureikat AH, Singhi AD, Boone B, Bahary N, Hogg ME, Lotze M, Zeh HJ III, Tublin ME. Assessment of Response to Neoadjuvant Therapy Using CT Texture Analysis in Patients With Resectable and Borderline Resectable Pancreatic Ductal Adenocarcinoma. AJR Am J Roentgenol. 2020 Feb;214(2):362-369. doi: 10.2214/AJR.19.21152. Epub 2019 Dec 4. PMID: 31799875; PMCID: PMC7457395.
63. Ren W, Xourafas D, Ashley SW, Clancy TE. Temporal Assessment of Prognostic Factors in Patients With Pancreatic Ductal Adenocarcinoma Undergoing Neoadjuvant Treatment and Resection. J Surg Res. 2021 Jan;257:605-615. doi: 10.1016/j.jss.2020.07.073. Epub 2020 Sep 15. PMID: 32947122.
64. Al Abbas AI, Zenati M, Reiser CJ, Hamad A, Jung JP, Zureikat AH, Zeh HJ 3rd, Hogg ME. Serum CA19-9 Response to Neoadjuvant Therapy Predicts Tumor Size Reduction and Survival in Pancreatic Adenocarcinoma. Ann Surg Oncol. 2020 Jun;27(6):2007-2014. doi: 10.1245/s10434-019-08156-3. Epub 2020 Jan 2. Erratum in: Ann Surg Oncol. 2020 Feb 7;: PMID: 31898105.

65. Vega EA, Kutlu OC, Salehi O, James D, Alarcon SV, Herrick B, Krishnan S, Kozyreva O, Conrad C. Preoperative Chemotherapy for Pancreatic Cancer Improves Survival and R0 Rate Even in Early Stage I. J Gastrointest Surg. 2020 Oct;24(10):2409-2415. doi: 10.1007/s11605-020-04601-x. Epub 2020 May 11. PMID: 32394126.

Outcomes pooled for patients receiving FOLFIRINOX and FOLFIRINOX + RTx, n=13

66. Yoo C, Kang J, Kim KP, Lee JL, Ryoo BY, Chang HM, et al. Efficacy and safety of neoadjuvant FOLFIRINOX for borderline resectable pancreatic adenocarcinoma: Improved efficacy compared with gemcitabine-based regimen. Oncotarget. 2017;8(28):46337-47.
67. Javed AA, Wright MJ, Siddique A, Blair AB, Ding D, Burkhart RA, et al. Outcome of Patients with Borderline Resectable Pancreatic Cancer in the Contemporary Era of Neoadjuvant Chemotherapy. J Gastrointest Surg. 2019;23(1):112-21.
68. Kim HS, Jang JY, Han Y, Lee KB, Joo I, Lee DH, et al. Survival outcome and prognostic factors of neoadjuvant treatment followed by resection for borderline resectable pancreatic cancer. Ann surg treat res. 2017;93(4):186-94.
69. Boone BA, Steve J, Krasinskas AM, Zureikat AH, Lembersky BC, Gibson MK, et al. Outcomes with FOLFIRINOX for borderline resectable and locally unresectable pancreatic cancer. J Surg Oncol. 2013;108(4):236-41.
70. Byun Y, Han Y, Kang JS, Choi YJ, Kim H, Kwon W, et al. Role of surgical resection in the era of FOLFIRINOX for advanced pancreatic cancer. J Hepato-Biliary-Pancreatic Sci. 2019;26(9):416-25.
71. Grose D, McIntosh D, Jamieson N, Carter R, Dickson E, Chang D, et al. The role of induction chemotherapy + chemoradiotherapy in localised pancreatic cancer: Initial experience in Scotland. J Gastrointest Oncol. 2017;8(4):683-95.
72. Paniccia A, Edil BH, Schulick RD, Byers JT, Meguid C, Gajdos C, et al. Neoadjuvant FOLFIRINOX application in borderline resectable pancreatic adenocarcinoma: a retrospective cohort study. Medicine (Baltimore). 2014;93(27):e198.
73. Choi YJ, Byun Y, Kang JS, Kim HS, Han Y, Kim H, Kwon W, Oh DY, Paik WH, Lee SH, Ryu JK, Kim YT, Lee K, Kim H, Chie EK, Jang JY. Comparison of Clinical Outcomes of Borderline Resectable Pancreatic Cancer According to the Neoadjuvant Chemo-Regimens: Gemcitabine versus FOLFIRINOX. Gut Liver. 2020 Aug 26. doi: 10.5009/gnl20070. Epub ahead of print. PMID: 32839360.
74. Macedo FI, Ryon E, Maithel SK, Lee RM, Kooby DA, Fields RC, Hawkins WG, Williams G, Maduekwe U, Kim HJ, Ahmad SA, Patel SH, Abbott DE, Schwartz P, Weber SM, Scoggins CR, Martin RCG, Dudeja V, Franceschi D, Livingstone AS, Merchant NB. Survival Outcomes Associated With Clinical and Pathological Response Following Neoadjuvant FOLFIRINOX or Gemcitabine/Nab-Paclitaxel Chemotherapy in Resected Pancreatic Cancer. Ann Surg. 2019 Sep;270(3):400-413. doi: 10.1097/SLA.0000000000003468. PMID: 31283563.
75. Paniccia A, Edil BH, Schulick RD, Byers JT, Meguid C, Gajdos C, McCarter MD. Neoadjuvant FOLFIRINOX application in borderline resectable pancreatic adenocarcinoma: a retrospective cohort study. Medicine (Baltimore). 2014 Dec;93(27):e198. doi: 10.1097/MD.0000000000000198. PMID: 25501072; PMCID: PMC4602784.
76. Templeton S, Moser M, Wall C, Shaw J, Chalchal H, Luo Y, Zaidi A, Ahmed S. Outcomes of Patients with Borderline Resectable Pancreatic Cancer Treated with Combination Chemotherapy. J Gastrointest Cancer. 2020 May 21. doi: 10.1007/s12029-020-00417-9. Epub ahead of print. PMID: 32440849.
77. Perri G, Prakash L, Qiao W, Varadhachary GR, Wolff R, Fogelman D, Overman M, Pant S, Javle M, Koay EJ, Herman J, Kim M, Ikoma N, Tzeng CW, Lee JE, Katz MHG. Response and Survival Associated With First-line FOLFIRINOX vs Gemcitabine and nab-Paclitaxel Chemotherapy for Localized Pancreatic Ductal Adenocarcinoma. JAMA Surg. 2020 Sep 1;155(9):832-839. doi: 10.1001/jamasurg.2020.2286. PMID: 32667641; PMCID: PMC7364337.

78. Garnier J, Ewald J, Marchese U, Gilabert M, Moureau-Zabotto L, Giovannini M, Poizat F, Delpero JR, Turrini O. Borderline or locally advanced pancreatic adenocarcinoma: A single center experience on the FOLFIRINOX induction regimen. Eur J Surg Oncol. 2020 Aug;46(8):1510-1515. doi: 10.1016/j.ejso.2020.02.037. Epub 2020 Feb 27. PMID: 32146053.
**Overlapping cohort with included studies, n=4**
79. Jang JK, Byun JH, Kang JH, Son JH, Kim JH, Lee SS, Kim HJ, Yoo C, Kim KP, Hong SM, Seo DW, Kim SC, Lee MG. CT-determined resectability of borderline resectable and unresectable pancreatic adenocarcinoma following FOLFIRINOX therapy. Eur Radiol. 2020 Aug 26. doi: 10.1007/s00330-020-07188-8. Epub ahead of print. PMID: 32845389.<br>80. Yoo C, Lee SS, Song KB, Jeong JH, Hyung J, Park DH, Song TJ, Seo DW, Lee SK, Kim MH, Lee SS, Kim JH, Jin HS, Park JH, Hwang DW, Lee JH, Lee W, Chang HM, Kim KP, Ryoo BY, Kim SC. Neoadjuvant modified FOLFIRINOX followed by postoperative gemcitabine in borderline resectable pancreatic adenocarcinoma: a Phase 2 study for clinical and biomarker analysis. Br J Cancer. 2020 Aug;123(3):362-368. doi: 10.1038/s41416-020-0867-x. Epub 2020 May 20. PMID: 32433600; PMCID: PMC7403346.<br>81. Neyaz A, Tabb ES, Shih A, Zhao Q, Shroff S, Taylor MS, Rickelt S, Wo JY, Fernandez-Del Castillo C, Qadan M, Hong TS, Lillemoe KD, Ting DT, Ferrone CR, Deshpande V. Pancreatic ductal adenocarcinoma: tumour regression grading following neoadjuvant FOLFIRINOX and radiation. Histopathology. 2020 Jul;77(1):35-45. doi: 10.1111/his.14086. Epub 2020 Jun 1. PMID: 32031712.<br>82. Golan T, Barenboim A, Lahat G, Nachmany I, Goykhman Y, Shacham-Shmueli E, Halpern N, Brazowski E, Geva R, Wolf I, Goldes Y, Ben-Haim M, Klausner JM, Lubezky N. Increased Rate of Complete Pathologic Response After Neoadjuvant FOLFIRINOX for BRCA Mutation Carriers with Borderline Resectable Pancreatic Cancer. Ann Surg Oncol. 2020 Oct;27(10):3963-3970. doi: 10.1245/s10434-020-08469-8. Epub 2020 Apr 20. PMID: 32314163.

Supplementary Figure 1. Funnel plot assessing risk of publication bias using primary outcome of overall survival.

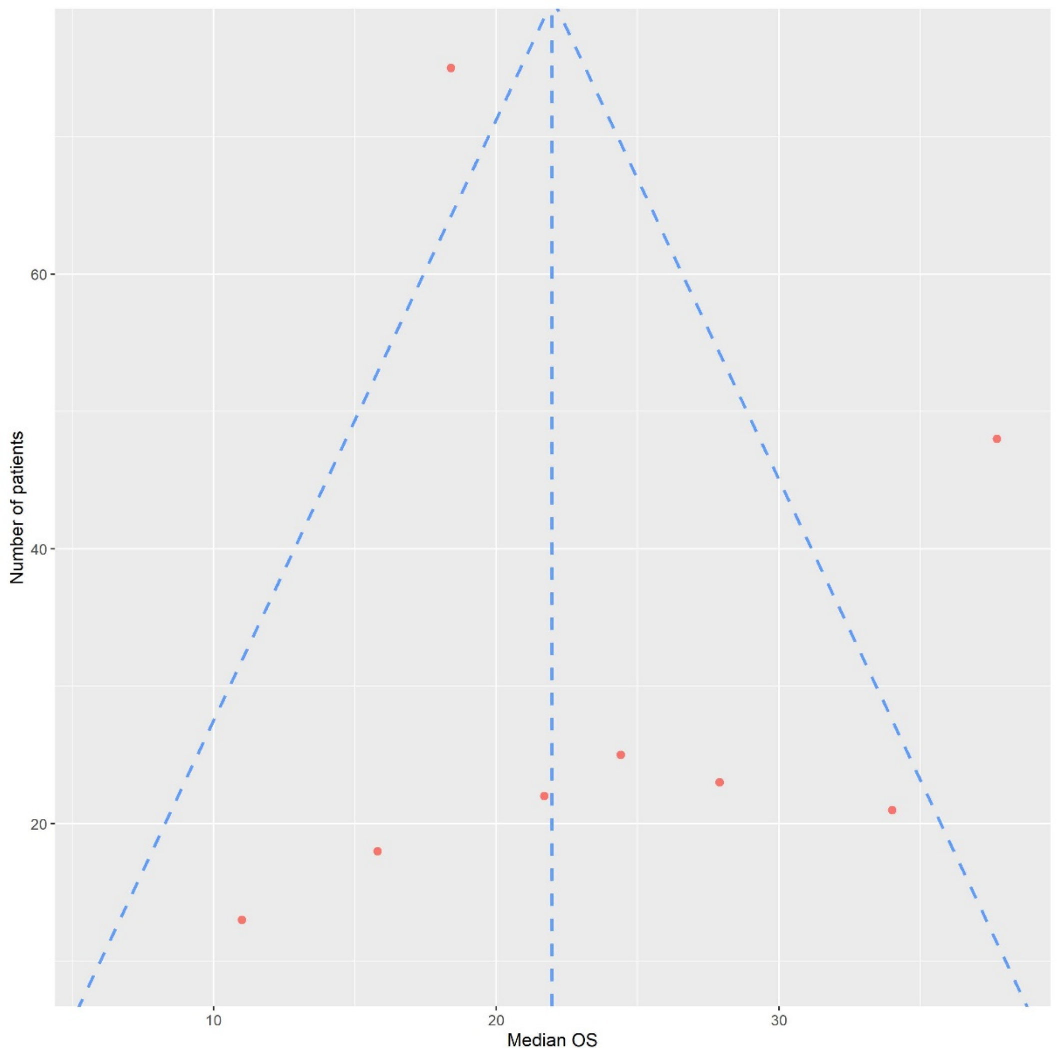

Supplement: Supplementary file 1 — Supplementary file1 (PDF 271 kb) [file 10434_2021_10276_MOESM1_ESM.pdf]
